# Fibrin sealant for prophylaxis of cervical anastomotic leakage after McKeown esophagectomy for esophageal or esophagogastric junction cancer (PLACE030): a Phase III multicenter randomized clinical trial

**DOI:** 10.1097/JS9.0000000000004514

**Published:** 2026-02-10

**Authors:** Zhichao Li, Geng Wang, Teng Mao, Lin Peng, Weidong Wang, Wenqiang Lv, Yan Huang, Caiyan Fang, Jiadi Wu, Jibin Li, Sharvesh Raj Seeruttun, Shaobin Chen, Yi Liang, Yu Yang, Chenghao Wang, Bin Li, Sheng Huang, Jiyang Chen, Huilin Su, Weizhao Huang, Ze-Rui Zhao, Hong Yang

**Affiliations:** a Department of Thoracic Surgery, State Key Laboratory of Oncology in South China, Collaborative Innovation Center for Cancer Medicine, Sun Yat-sen University Cancer Center, Guangzhou, Guangdong Province, China; b Department of Thoracic Surgery, Cancer Hospital of Shantou University Medical College, Shantou, Guangdong Province, China; c Department of Thoracic Surgery, Shanghai Chest Hospital, Shanghai Jiaotong University, Shanghai, China; d Sichuan Cancer Center, Department of Thoracic Surgery, Sichuan Cancer Hospital & Institute, School of Medicine, University of Electronic Science and Technology of China, Chengdu, Sichuan Province, China; e Department of Thoracic Surgery, People’s Hospital of Jieyang, Jieyang, Guangdong Province, China; f Department of Good Clinical Practice, State Key Laboratory of Oncology in South China, Collaborative Innovation Center for Cancer Medicine, Sun Yat-sen University Cancer Center, Guangzhou, Guangdong Province, China; g State Key Laboratory of Oncology in South China, Collaborative Innovation Center for Cancer Medicine, Sun Yat-sen University Cancer Center, Guangzhou, Guangdong Province, China; h Department of Cardiothoracic Surgery, Zhongshan Hospital of Sun Yat-Sen University (Zhongshan People’s Hospital), Zhongshan, Guangdong Province, China; i Department of Thoracic Surgery, The Second Hospital & Clinical Medical School, Lanzhou University, Lanzhou, Gansu Province, China; j Department of Thoracic Surgery, State Key Laboratory of Oncology in South China, Collaborative Innovation Center for Cancer Medicine, Guangdong Esophageal Cancer Institute, Sun Yat-sen University Cancer Center, Guangzhou, Guangdong Province, China

**Keywords:** anastomotic leakage, esophageal cancer, fibrin sealant, McKeown esophagectomy, postoperative complications

## Abstract

**Purpose:**

Anastomotic leakage (AL) is one of the most serious complications after esophagectomy, and previous studies have suggested that fibrin sealant (FS) might be beneficial in preventing AL. This study aimed to evaluate the efficacy and safety of FS in preventing AL.

**Methods:**

This randomized controlled trial included 360 patients aged 18-75 years with resectable esophageal or esophagogastric junction cancer, clinically staged as T1-4aN0-3M0, from six centers in China. Surgery was performed using McKeown esophagectomy with circular stapled anastomosis combined with two-field lymphadenectomy. In the FS group, 2.5 ml of FS was applied circumferentially to the cervical anastomotic site. The primary endpoint was the incidence of AL within the first 3 months postoperatively.

**Results:**

A total of 360 patients were recruited from February 2019 to May 2023, with 179 in the FS group and 181 in the control group. The incidence of AL was 7.3% in the FS group and 13.3% in the control group (*P* = 0.061). According to the subgroup analysis of patients who underwent upfront surgery without neoadjuvant treatment, the incidence of AL in the FS group was numerically lower without statistical significance [FS group: 5.8% vs. control group: 15.1%; *P* = 0.046, RR = 0.385 (95% CI, 0.143-1.032)]. The incidence of postoperative complications was similar between the two groups (FS group: 41.6% vs. control group: 48.9%; *P* = 0.163).

**Conclusions:**

Intraoperative sealing with porcine FS did not significantly reduce the incidence of cervical AL. For patients who undergo upfront esophagectomy, the potential protective effect of FS needs further studies.

## Introduction

Esophageal cancer (EC) is the eleventh most commonly diagnosed cancer and the seventh leading cause of cancer-related death globally, with approximately 511,000 new cases and 445,000 deaths reported in 2022^[1]^. The incidence and mortality rates in China account for over half of the global burden^[1,2]^. Currently, surgery is the primary treatment for EC or esophagogastric junction cancer (EJC). Among the surgical options, McKeown esophagectomy is the most frequently performed procedure, with this technique being favored for its capability to achieve adequate resection and comprehensive lymphadenectomy^[3]^. However, for cervical anastomosis, McKeown esophagectomy is generally associated with a higher risk of postoperative complications than procedures involving intrathoracic anastomosis, especially in terms of anastomotic leakage (AL)^[4,5]^.

As one of the most severe postoperative complications, AL has a high incidence, ranging from 10.6%-26.1% worldwide, leading to prolonged hospital stays, increased hospitalization costs, and high probabilities of morbidity and mortality^[5–10]^. Multiple anastomosis methods, including the application of fibrin sealant (FS) to the anastomosis intraoperatively, have been developed to prevent AL after esophagectomy. The inclusion of thrombin and fibrinogen in FS functions through several key mechanisms, such as expediting the coagulation cascade, modulating leukocyte populations and inflammatory responses, and enhancing granulation maturation. FS can promote anastomotic healing not only by blocking tissue defects but also by forming a fibrin matrix where fibroblasts and capillary endothelial cell proliferation occur, enabling granulation tissue formation^[11,12]^. Thus far, several clinical studies have indicated that the application of FS might be beneficial for the prevention of AL. However, due to the lack of randomized controlled trials, there is insufficient evidence to prove the efficacy of FS for preventing cervical AL^[13–17]^. A well-designed, large-scale, randomized controlled trial is needed to evaluate the usefulness of FS.

The current phase III trial included patients who underwent McKeown surgery for EC or EJC. The primary purpose of this study was to evaluate the efficacy and safety of porcine FS for preventing AL. This article is compliant with the TITAN Guidelines 2025 – governing declaration and use of AI^[18]^.

## Patients and methods

### Eligibility

Eligible patients had histologically confirmed resectable squamous cell carcinoma or adenocarcinoma of the thoracic esophagus or esophagogastric junction, staged as T1-4aN0-3M0 according to the 8th AJCC edition. They were between 18 and 75 years of age, had normal liver and kidney functions, a WHO performance status score of 0-1, and an expected survival time of more than 6 months. Exclusion criteria included: (1) cervical EC; (2) surgical contraindications due to cardiac, respiratory, hepatic, renal diseases, or other uncontrollable conditions; (3) prior gastrectomy making stomach conduit reconstruction infeasible; (4) prior definitive chemoradiotherapy; (5) history of or concomitant hemorrhagic diseases; (6) diabetes with poor glycemic control lasting more than 10 years; (7) known hypersensitivity to porcine FS products; and (8) pregnancy or lactation (Supplemental Digital Content Supplement 1, available at: http://links.lww.com/JS9/G884).

This study was approved by the ethics committee or institutional review board at each center. All patients provided written informed consent. This trial was registered at ClinicalTrials.gov. The work has been reported in line with Consolidated Standards of Reporting Trials (CONSORT) Guidelines^[19]^.

**HIGHLIGHTS**

- This phase III trial assessed porcine fibrin sealant (FS) for preventing anastomotic leakage (AL) after McKeown esophagectomy.
- FS application did not significantly reduce overall AL rates compared to standard care (7.3% vs. 13.3%, *P* = 0.061).
- A subgroup analysis highlighted a lower, albeit non-significant, AL incidence in the FS group among patients undergoing surgery without neoadjuvant treatment [5.8% vs. 15.1%; RR = 0.385 (95% CI, 0.143-1.032)].
- FS use may be unwarranted in patients receiving neoadjuvant treatment, aiding clinical decision-making and health economic burden reduction.

### Random assignment

The PLACE030 trial was a multicenter, prospective, randomized controlled trial involving 360 patients across six centers. Patients were randomized 1:1 into two groups using a stratified permuted-block method. One group received surgery plus the application of FS (FS group), while the other received surgery alone (control group). Random assignments were generated using computer-generated lists. Investigators at each center enrolled participants and assigned interventions. For the FS group, 2.5 ml of FS (BioSeal; Guangzhou Bioseal Biotech Co., Ltd., China) was applied to cover the full circumference of the cervical anastomotic site. The effectiveness and safety of Bioseal for preventing AL were evaluated (Supplemental Digital Content Supplementary Materials Protocol 2, available at: http://links.lww.com/JS9/G88).

### Pretreatment workup and staging

All patients underwent pretreatment examinations and staging, including chest and abdominal contrasted computed tomography (CT), esophagogastroduodenoscopy (EGD) with endoscopic ultrasonography, electrocardiogram, lung spirometry, and cervical ultrasonography. Bronchoscopy was performed, if indicated, to exclude tumor infiltration into the trachea or bronchial tree. Positron emission tomography and radionuclide bone imaging were optional.

### Surgery

**Figure 1.**
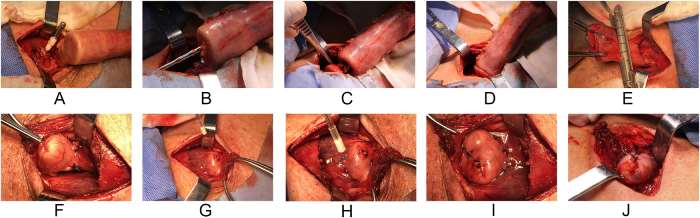
Step-by-step procedure of circular stapled anastomosis using fibrin sealant in esophagogastric anastomosis. **(A)** The stapler head is carefully inserted into the cervical esophageal stump and securely fastened. (**B**) The gastric wall is incised, and the stapler is inserted. (C) An esophagogastric end-to-side anastomosis is performed using a circular stapler. (D) Completion of the end-to-side anastomosis. (E) The fundus of the gastric conduit is closed using a linear stapler. (F) The staple line is sutured inward for reinforcement. (G-H) Fibrin sealant is applied circumferentially to the esophagogastric anastomosis. (I-J) Completion of the fibrin sealant application to the cervical anastomosis.

The surgical procedures were performed by consultant surgeons at each center. McKeown esophagectomy, including two-field lymphadenectomy with total mediastinal lymph node dissection, was performed through thoracotomy or a minimally invasive approach. Total lymphadenectomy involved resection of the following lymph node stations: bilateral recurrent laryngeal nerve nodes, periesophageal nodes, subcarinal nodes, pulmonary ligament nodes, diaphragmatic nodes, pericardiac nodes, lesser curvature nodes, left gastric nodes, common hepatic nodes, splenic nodes, and celiac nodes. An end-to-side cervical esophagogastric anastomosis was constructed via a circular stapler (21 or 25 mm, Covidien, USA; 21 or 25 mm, Ethicon, USA) and additionally wrapped with interrupted sutures for reinforcement. Then, we closed the gastric remnant using a linear stapler (Endo GIA, Covidien, USA; or Echelon Flex, Ethicon, USA) and wrapped it with seromuscular-layer interrupted sutures. Two point five ml of Bioseal [containing two main components: fibrinogen (≥30.0 mg/Ml) and thrombin (450-850 IU/mL)] was irrigated over the cervical anastomotic site for patients in the FS group. The specific anastomotic technique utilized for the intraoperative application of fibrin sealant to the cervical anastomosis is depicted in Fig. 1. Subsequently, a nasogastric tube and a cervical drainage tube were placed. Additionally, either a jejunal nutrition tube was placed by jejunostomy or a nasal feeding nutritional tube was placed for the postoperative provision of nutrition.

Postoperatively, patients were routinely transferred to the intensive care unit (ICU) on the first day and then readmitted to the general ward on the second day when appropriate. An EGD or esophageal barium X-ray was routinely performed on the seventh postoperative day if feasible. Oral feeding commenced following the elimination of AL.

### Pathologic analysis

Pathologic examination reports included tumor type, extent, proximal and distal resection margins, tumor regression grade, and lymph node status, specifying the site and number of nodes with therapeutic effects. Pathologic complete response (pCR) was defined as no evidence of residual tumor cells in the primary site or in the resected lymph nodes of the operative specimens.

### Outcomes

The primary endpoint was the incidence of cervical AL within the first 3 months after the operation. Cervical AL was defined as a full-thickness gastrointestinal defect involving the esophagus, anastomosis, or gastric stump. The diagnostic criteria for AL were as follows: macroscopic anastomotic dehiscence or fistula that was visible through EGD; barium leakage shown by esophageal barium X-ray; digestive fluid leakage from the chest tube or cervical wound; and methylene blue dye leakage through the cervical wound or chest tube after oral administration. EGD or esophageal barium X-ray was routinely performed on each individual on the seventh postoperative day if feasible.

The secondary endpoints included postoperative anastomotic stricture incidence (diagnosed by dysphagia and an anastomotic orifice diameter ≤10 mm on esophageal barium examination), postoperative morbidity and mortality rates, time to first oral feeding, 2-year overall survival rate, and disease-free survival rate. Postoperative complications were categorized as minor complications (grades I and II) and major complications (grades III, IV, and V) based on the Clavien–Dindo classification of surgical complications.

### Follow-up

Posttreatment follow-up included outpatient examinations every 3 months for the first year and every 6 months thereafter until death. Follow-up visits involved physical examination, routine blood tests, tumor marker analysis, chest X-ray, esophageal barium X-ray, and cervical and abdominal ultrasonography. Contrast-enhanced cervicothoracoabdominal CT and EGD were performed annually.

### Statistical analysis

Intention-to-treat analyses were conducted for this study. Subgroup analyses by age, sex, body mass index (BMI), tumor location, clinical stage and neoadjuvant therapy were prespecified. On the basis of our phase II study and other previous studies, sample size calculations were made assuming a projected cervical anastomotic leakage rate of 5% and 15% for the FS group and control group, respectively. With an overall two-sided significance level of 5% and a statistical power of 80%, a randomization ratio of 1:1 between the FS group and control group, 4 years of accrual, 2 years of follow-up, and one planned interim analysis, and with a 10% dropout rate accounted for, the intended number of randomly assigned patients was 360 (180 per arm).

Following the enrolment of 180 patients (50%), a planned interim analysis was conducted. According to the O’Brien‒Fleming algorithm, the two-sided significance level was 0.005 in the interim analysis and 0.048 in the final analysis. The postoperative cervical AL rate, anastomotic stricture rate, morbidity, and mortality rate were calculated as percentages and compared by Pearson’s chi-squared test or Fisher’s exact test. Student’s t test and the Mann–Whitney U test were used for comparison of continuous variables, such as the number of dissected lymph nodes, operative time and blood loss volume. Age, sex, BMI, tumor location, clinical stage, application of FS, and neoadjuvant therapy were included in the multivariate model after the study was completed to identify variables associated with AL. Logistic regression analysis was used to define differences in the incidence of AL between groups and subgroups. All the statistical analyses were performed using SPSS 22.0 (IBM Corporation, USA).

## Results

### Baseline characteristics

**Figure 2.**
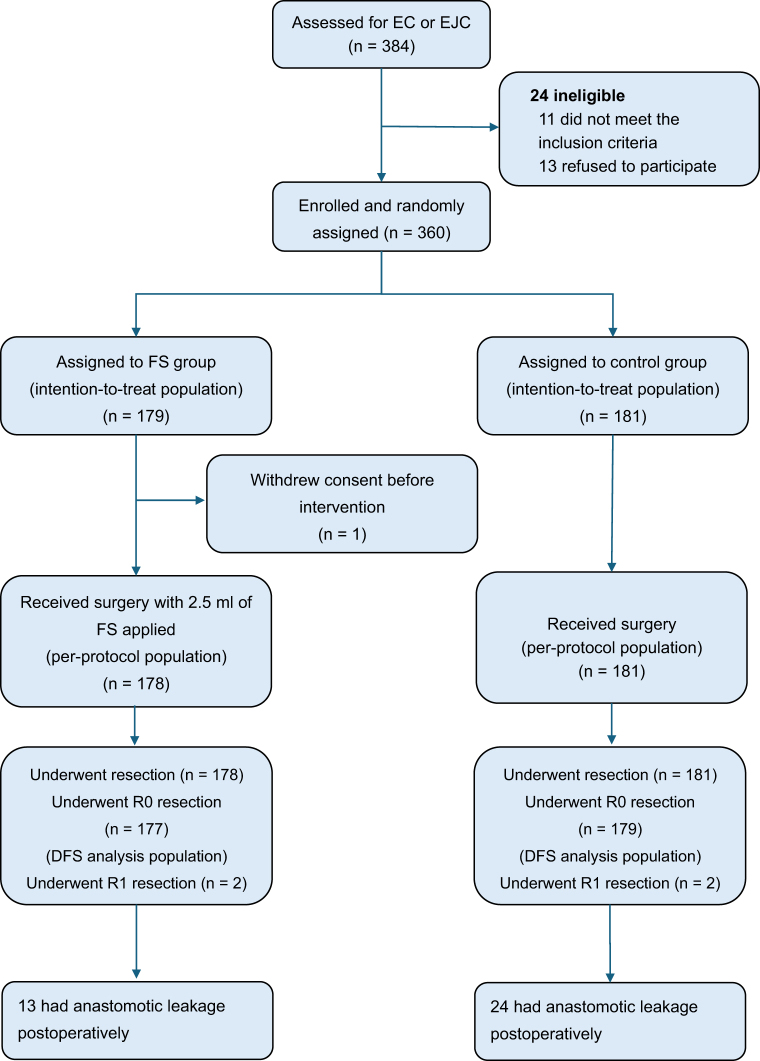
Consort diagram. EC, esophageal cancer; EJC, esophagogastric junction cancer.

From February 2019 to May 2023, a total of 360 patients from six Chinese centers were randomly allocated to the FS group (n = 179) or the control group (n = 181; Fig. 2). Table 1 summarized the baseline characteristics of the two groups, which were well-balanced. Most of the patients who were enrolled were male (80.3%), had tumors located in the thoracic region (94.4%), and had squamous cell carcinoma (94.4%). The percentage of patients who received neoadjuvant therapy was similar in the two groups (FS group: 52.0% vs. control group: 52.5%; *P* = 0.920).

**Table 1:** Baseline characteristics of patients in fibrin sealant and control groups.

Characteristics	FS group, no. (%) (n = 179)	Control group, no. (%) (n = 181)	*P*-value -
Age, years			0.740
Median (range)	62.0 (38.0, 75.0)	62.0 (33.0, 75.0)	
≤60	77 (43.0)	81 (44.8)	
>60	102 (57.0)	100 (55.2)	
Sex			0.542
Male	146 (81.6)	143 (79.0)	
Female	33 (18.4)	38 (21.0)	
BMI			0.592
BMI < 24	127 (70.9)	133 (73.5)	
BMI ≥ 24	52 (29.1)	48 (26.5)	
Location of tumor			0.942^a^
Upper	15 (8.4)	18 (9.9)	
Middle	80 (44.7)	74 (40.9)	
Lower	75 (41.9)	78 (43.1)	
Esophagogastric junction	6 (3.3)	7 (3.9)	
Multiple primary tumor	3 (1.7)	4 (2.2)	
Surgical approach			0.170^a^
Open esophagectomy	9 (5.0)	4 (2.2)	
MIE	170 (95.0)	177 (97.8)	
Histology			0.766^a^
Squamous cell carcinoma	169 (94.4)	171 (94.5)	
Adenocarcinoma	8 (4.5)	6 (3.3)	
Adenosquamous carcinoma	0	1 (0.6)	
Other	2 (1.1)	3 (1.7)	
Pretreatment stage			
Clinical T stage			0.836
cT1	19 (10.6)	17 (9.4)	
cT2	42 (23.5)	45 (24.9)	
cT3	104 (58.1)	101 (55.8)	
cT4	14 (7.8)	18 (9.9)	
Clinical N stage			0.848
N0	76 (42.5)	69 (38.1)	
N1	53 (29.6)	56 (30.9)	
N2	38 (21.2)	44 (24.3)	
N3	12 (6.7)	12 (6.6)	
Clinical stage			0.865
I	19 (10.6)	17 (9.4)	
II	66 (36.9)	62 (34.3)	
III	65 (36.3)	74 (40.9)	
IVA	29 (16.2)	28 (15.5)	
Neoadjuvant therapy			0.925
None	86 (48.0)	86 (47.5)	
Chemoradiotherapy	56 (31.3)	60 (33.1)	
Chemotherapy	37 (20.7)	35 (19.3)	

BMI body mass index, MIE minimally invasive esophagectomy

^a^ Using Fisher’s exact test

### Surgery

Of the 360 patients, the majority underwent McKeown esophagectomy via Minimally Invasive Esophagectomy (FS group: 95.0% vs. control group: 97.8%; *P* = 0.170). Among the 179 patients in the FS group, 177 (98.9%) achieved R0 resection, compared to 179 of 181 patients (98.9%) in the control group (*P* = 1.00, Table 2). R1 resection was observed in two patients in each group due to microscopically positive margins. The median number of resected lymph nodes was 27 [interquartile range (IQR), 21-35] in the FS group and 26 (IQR, 20-35) in the control group (*P* = 0.429). The median operating time was similar between the two groups [FS group: 240.5 (IQR, 204.8-276.3) minutes vs. control group: 247.0 (IQR, 210.0-285.5) minutes; *P* = 0.378], and the estimated blood loss was also comparable [FS group: 50.0 (IQR, 50.0-100.0) ml vs. control group: 50.0 (IQR, 50.0-100.0) ml; *P* = 0.799].

**Table 2:** Intraoperative outcomes comparing fibrin sealant application with control.

Outcomes	FS group, (n = 179)	Control group, (n = 181)	*P*-value -
Radicality of surgery			1.000^a^
R0	177 (98.9)	179 (98.9)	
R1	2 (1.1)	2 (1)	
Number of LNs (median [IQR])	27 (21-35)	26 (20-35)	0.429
Operative time (median [IQR])	240.5 (204.8-276.3)	247.0 (210.0-285.5)	0.378
Blood loss (median [IQR])	50.0 (50.0-100.0)	50.0 (50.0-100.0)	0.799

^a^ Using Fisher’s exact test

### Histopathological results

With respect to the distribution of pathologic stage, most patients who underwent surgery alone had stage II-III disease (65.1%), while patients with pathological stage I disease (57.4%) comprised the largest proportion of those who received neoadjuvant therapy (Table 3). Among the patients receiving neoadjuvant therapy, the pCR rate was 39.9% (37/93) in the FS group and 40.0% (38/95) in the control group (*P* = 0.983). No significant difference was found in the downstaging rate between the two groups (FS group: 74.5% vs. control group: 75.8%; *P* = 0.967).

**Table 3:** Distribution of pathological stages in patients undergoing surgery alone or with neoadjuvant therapy.

Pathological stage	FS group, (n = 179)	Control group, (n = 181)	*P*-value
Pathological T stage			0.461^a^
pT0	46 (25.7)	51 (28.2)	
pT1	45 (25.1)	31 (17.1)	
pT2	28 (15.6)	34 (18.8)	
pT3	58 (32.4)	63 (34.8)	
pT4	2 (1.1)	2 (1.1)	
Pathological N stage			0.717^a^
pN0	117 (65.4)	120 (66.3)	
pN1	43 (24.0)	48 (26.5)	
pN2	16 (8.9)	11 (6.1)	
pN3	3 (1.7)	2 (1.1)	
Pathological stage			
Surgery alone, no. (%)	n = 86	n = 86	0.694^a^
Tis	4 (4.7)	4 (4.7)	
I	26 (30.2)	20 (23.3)	
II	28 (32.6)	30 (34.9)	
III	24 (27.9)	30 (34.9)	
IVa	4 (4.7)	2 (2.3)	
Neoadjuvant therapy, no. (%)	n = 93	n = 95	0.572^a^
I	53 (57.0)	55 (57.9)	
II	10 (10.8)	14 (14.7)	
III	30 (32.3)	25 (26.3)	
IVa	0	1 (1.1)	
pCR	37 (39.9)	38 (40.0)	

^a^ Using Fisher’s exact test

### Anastomotic leakage

**Figure 3.**
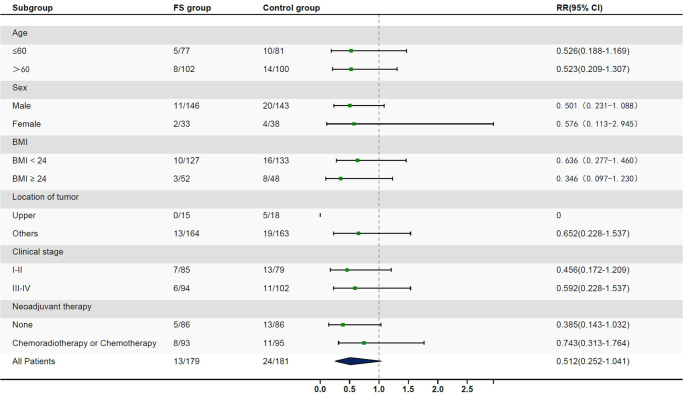
Subgroup analysis of anastomotic leakage incidence.

By December 2024, the median follow-up time was 29.7 months (IQR, 22.4-39.6 months) in the FS group and 30.5 months (IQR, 23.9-41.3 months) in the control group. The incidence of AL was 7.3% (95% CI, 3.9-12.2) in the FS group and 13.3% (95% CI, 8.6-19.0) in the control group, and no significant difference was detected (*P* = 0.061, Table 4). According to the subgroup analysis, patients who underwent upfront surgery showed a nonsignificant trend toward better outcomes with the application of FS (Fig. 3). The incidence of AL in the FS group was numerically lower without statistical significance [FS group: 5.8% (95% CI, 1.9-13.2) vs. control group: 15.1% (95% CI, 8.2-24.2); *P* = 0.046, relative risk (RR) = 0.385 (95% CI, 0.143-1.032)] among these patients. Moreover, for patients who received neoadjuvant therapy plus surgery, the incidence of AL was comparable between the two groups [FS group: 8.6% (95% CI, 3.8-16.2) vs. control group: 11.6% (95% CI, 5.9-19.8); *P* = 0.498, RR = 0.743 (95% CI, 0.313-1.764)]. Among the patients who had cervical AL, one patient in each group was diagnosed with a gastric stump fistula and underwent a subsequent surgical procedure to reconstruct the gastric stump. All the other patients with AL were successfully managed after conservative treatment with fasting, cervical dressing, and endoscopic treatment.

**Table 4:** Incidence of postoperative complications in fibrin sealant and control groups within 3 months.

Complications	FS group, no.(%)	Control group, no.(%)	P-value
	(n = 179)	(n = 181)	
Cervical anastomotic leakage	13 (7.3)	24 (13.3)	0.061
Anastomotic stricture	13 (7.3)	8 (4.4)	0.250
Thoracic gastric fistula	1 (0.6)	0	0.497^a^
Pneumonia	15 (8.4)	29 (16.0)	0.027
Arrhythmia	6 (3.4)	9 (5.0)	0.442
Heart failure	5 (2.8)	5 (2.8)	0.986
Respiratory failure	8 (4.5)	12 (6.6)	0.371
Pneumothorax	5 (2.8)	4 (2.2)	0.750^a^
Laryngeal nerve injury	13 (7.3)	16 (8.8)	0.582
ARDS	0	6 (3.3)	0.030^a^
Atelectasis	1 (0.6)	4 (2.2)	0.372^a^
Pyothorax	2 (1.1)	2 (1.1)	1.000^a^
Chylothorax	0	1 (0.6)	1.000^a^
Minor complications	40 (22.3)	43 (23.8)	0.751
Major complications	36(20.1)	44 (24.3)	0.338

^a^ Using Fisher’s exact test

According to both the univariate and multivariate analyses, FS tended to be a protective factor for AL, although the difference was not statistically significant [univariate analysis: odds ratio (OR) 0.519 (95% CI, 0.255-1.054), *P* = 0.070; multivariate analysis: OR 0.505 (95% CI, 0.248-1.031), *P* = 0.061] (Table 5). Additionally, logistic regression analyses did not identify any specific variables significantly associated with AL.

**Table 5:** Logistic regression analysis of factors associated with anastomotic leakage.

Variables	Univariate analyses			Multivariate analyses		
	OR	95% CI	*P*	OR	95% CI	*P*
Sex (male vs. female)	1.302	0.521-3.252	0.572	1.384	0542-3.535	0.496
Age (>60 vs. ≤ 60)	1.165	0.583-2.328	0.665	1.165	0.574-2.365	0.672
BMI (≥24 vs. < 24)	1.112	0.528-2.346	0.780	1.110	0.521-2.365	0.787
Location of tumor	0.813	0.522-1.266	0.359	0.810	0.513-1.280	0.367
Clinical stage	0.684	0.345-1.353	0.275	0.740	0.449-1.219	0.237
FS group vs. Control group	0.519	0.255-1.054	0.070	0.505	0.248-1.031	0.061
Neoadjuvant therapy vs. None	1.040	0.526-2.053	0.911	1.291	0.543-3.068	0.563

### Other morbidities

The postoperative complications are summarized in Table 4. Of the 360 patients, 163 (45.2%) developed postoperative complications, including 80 (22.2%) with major complications and 83 (23.1%) with minor complications. Postoperative complications did not differ significantly between the two groups, with the exception of pneumonia (*P* = 0.027) and ARDS (*P* = 0.030). In addition to cervical AL, the most frequent complications were pneumonia and anastomotic stricture. The incidence of pneumonia was higher in the control group (16.0%) compared to the FS group (8.4%, *P* = 0.027). The rate of anastomotic stricture was 7.3% in the FS group and 4.4% in the control group, but this difference was not statistically significant (*P* = 0.250). One patient (0.6%) in the FS group developed an intrathoracic gastric conduit fistula and required conservative treatment, recovering 93 days postoperatively. There was no significant difference in the rate of recurrent laryngeal nerve injury between the FS group (7.3%) and the control group (8.8%, *P* = 0.582). No FS-related adverse event was reported within 90 days after surgery.

The median duration of hospitalization was similar between the two groups: 11 days (IQR, 9-17) for the FS group and 11 days (IQR, 9-20) for the control group (*P* = 0.675). Similarly, the median duration of ICU stay was comparable: 1 day (IQR, 1-2) for the FS group vs. 1 day (IQR, 1-4) for the control group (*P* = 0.602). The time to initiation of postoperative oral feeding did not differ significantly between the two groups [FS group: 8 days (IQR, 7-13) vs. control group: 8 days (IQR, 7-14), *P* = 0.744].

### Overall survival

**Figure 4.**
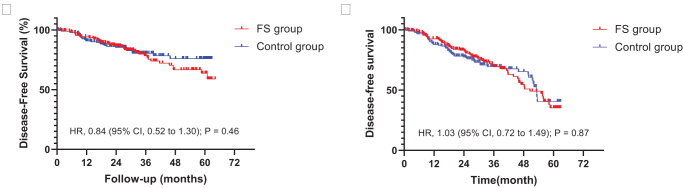
Overall survival and disease-free survival. (A) Overall survival in the intention-to-treat population. (B) Disease-free survival for patients after R0 resection.

Overall survival was not significantly different among patients in the FS group compared with those in the control group (HR = 0.84; 95% CI, 0.52-1.30; *P* = 0.46) (Fig. 4A). Overall survival rates at 2 years were 86.3% (95% CI, 79.1%-95.7%) in the FS group compared with 85.4% (95% CI, 81.2%-96.0%) in the control group. Disease-free survival was not significantly different between the two groups (HR = 1.03; 95% CI, 0.72-1.49; *P* = 0.87) (Fig. 4B).

## Discussion

This phase III clinical trial evaluated the efficacy of FS in preventing cervical AL following McKeown esophagectomy in patients with resectable thoracic EC or EJC. After a median follow-up of 30.1 months, no significant difference was observed in the incidence of cervical AL between the two groups. However, FS demonstrated a tendency to reduce the rate of AL in patients who underwent upfront surgery [5.8% vs. 15.1%, RR = 0.385 (95% CI, 0.143-1.032)].

The treatment protocol used in this study was the same as that used in our previous phase II trial^[20]^. In that study, only 3 (5.3%) of the 57 patients who underwent McKeown esophagectomy developed AL after the application of FS. The incidence of anastomotic stricture and other major postoperative complications was 1.8% and 17.5%, respectively. The median time to oral feeding initiation after the operation was 8 days (IQR 7.0-9.0). No adverse event related to FS was observed, and no death occurred within 90 days after surgery. On the basis of these results, the current study was conducted.

This study was designed to detect a 10% difference in the AL rate in favor of FS compared to the control group (5% vs. 15%). The final results indicated that the incidence of AL after esophagectomy in the control group was 13.3%, which is in line with the literature^[21]^, whereas the AL rate in the FS group was 7.3%, slightly higher than anticipated. Several factors contributed to the control of bias in this study. First, all patients underwent McKeown esophagectomy with esophagogastric anastomosis in the left cervical region. This approach is common in East Asia and is associated with a histologic predominance of squamous cell carcinoma in this cohort. Second, all anastomoses were performed using the circular stapling technique in the current study. According to prospective observational data from the Esophago-Gastric Anastomosis Audit study group^[22]^, the incidence of AL significantly differed among various anastomotic methods, from 19.3% in hand-sewn anastomoses to 14.0% and 12.1% in linear stapled and circular stapled anastomoses, respectively. Hence, the utility of FS can be discussed without the potential influence of different anastomotic techniques in this study. Third, total mediastinal lymph node dissection, especially recurrent laryngeal nerve node dissection, was implemented in the present trial. Finally, esophagogastroduodenoscopy or esophageal barium X-ray was performed to evaluate the status of the cervical anastomosis on postoperative day 7 if feasible.

Fibrin glue, which contains thrombin and fibrinogen, is advantageous for accelerating the coagulation cascade, modulating leukocyte populations and inflammatory responses and increasing granulation maturation^[11,12,23]^. Because FS can cover the microleakage and provide a scaffold for fibroblasts and capillary endothelial cells, its application is favored for preventing AL^[17]^. A recent systematic review and meta-analysis by Cira K. et al., encompassing 15 studies, found that collagen-based laminar biomaterials or FS significantly reduce the postoperative incidence of intestinal AL (OR = 0.37; 95% CI, 0.27-0.52)^[24]^. In the context of esophageal disease, a pilot study by Upadhyaya *et al* demonstrated that FS application might reduce AL rates in neonates with esophageal atresia following esophagectomy, with AL rates of 9.9% (2/22) in the FS group compared to 43% (10/23) in the control group^[13]^. For pediatric patients with caustic esophageal injury requiring colon interposition, Saldaña-Cortés *et al* reported AL rates of 28.5% (4/15) with FS application versus 50% (12/24) without FS^[14]^. In adult patients, Haverkamp *et al* reported the use of FS in 11 patients who underwent thoraco-laparoscopic esophagectomy with esophagogastrostomy. Two out of eleven patients (18.2%) required reoperation due to AL^[15]^. In another study, Plat *et al* reported the feasibility of FS for esophageal anastomosis reinforcement, with only 1 (6.7%) of the 15 patients developing AL^[16]^. However, the sample sizes in these studies were relatively small. In addition, these studies had some limitations, including the limited number of patients who received neoadjuvant treatment. Moreover, some esophagogastric anastomoses were performed intrathoracically in these studies, of which the AL rate was generally lower than that in McKeown esophagectomy. To our knowledge, the PLACE030 trial is the first large-scale, randomized controlled phase III trial to investigate the efficacy of FS for preventing cervical AL. It is noteworthy that all participants in this study were diagnosed with EC or EJC, distinguishing this research from most of the prior studies. Meanwhile, 52.2% of the participants in this trial received neoadjuvant treatment and 47.8% of the participants received upfront esophagectomy, indicating that further analysis of how preoperative therapy impacts the effectiveness of FS is feasible.

According to the subgroup analysis of patients who underwent upfront surgery, the incidence of AL was numerically lower in the FS group compared to the control group [5.8% vs. 15.1%, RR = 0.385 (95% CI, 0.143-1.032)]. This absolute difference of 9.3% is close to the trial’s initial assumption that FS may decrease AL incidence by 10%, and the statistical power was less than robust due to the small number of participants (n = 172). However, the AL rate did not differ among patients who received preoperative treatment. An experimental rat study by Verhage *et al* indicated that FS provides additional mechanical support, with measurements showing the highest bursting anastomotic pressures on postoperative days 0 and 3, when anastomotic strength was at its weakest^[25]^. On the other hand, complete healing largely depends on the sequential processes of neovascularization and proliferation of fibroblasts in the provisional fibrin matrix, degradation of the fibrin matrix, and the formation of tissue into a collagen-rich matrix^[23,26]^. The potential factors resulting in diminished healing impact following FS application in patients who underwent preoperative treatment can be succinctly summarized as several key points. First, chemotherapy and radiotherapy have the capacity to impede cellular proliferation, with fibroblasts and capillary endothelial cells being no exception, which directly impairs the wound healing capacity of these patients^[27]^. Additionally, the cytotoxic effects of these treatments not only reduce cell numbers but also alter the extracellular matrix composition, further undermining the structural integrity required for effective healing. Second, previous studies have suggested that either chemotherapy or irradiation inhibits neovascularization *in vivo* and results in diminished blood flow or vascular supply to the affected area, thereby impeding the healing process at the anastomotic site by restricting the delivery of oxygen and essential nutrients which is necessary for tissue repair and regeneration. This reduction in perfusion is particularly critical in the context of fibrin sealant application, as it operates within a biological environment that heavily relies on adequate blood flow to support the sequence of coagulation, fibroblast migration, and collagen deposition. Given that fibrin sealant depends on the body’s innate healing mechanisms to fortify the anastomotic seal, a compromised vascular supply may compromise its efficacy in facilitating healing, which ultimately leads to insufficient reconstitution of connective tissue^[27,28]^. Third, preoperative chemotherapy or chemoradiation can jeopardize blood flow to small branches of the gastroepiploic artery and thus affect the blood supply of the stomach, which might prolong the healing process of esophagogastric anastomosis^[29,30]^. Such vascular compromise may be exacerbated in patients with pre-existing microangiopathy or other comorbidities that further diminish tissue resilience and reparative capacity. In our practice, clinically involved lymph nodes in the gastric fundus can be included in the gross target volume for preoperative radiation. As a result, it could cause vessel obliteration and possibly ultimately hamper the formation of granulation tissue. The combined impact of radiation-induced fibrosis and localized ischemia creates a challenging microenvironment for healing, which even adjunctive measures like FS may not sufficiently overcome. Therefore, the healing impact following FS application could be counteracted by chemotherapy and radiotherapy.

Despite the significant role of neoadjuvant treatment in the comprehensive management of EC, a substantial proportion of patients with early-stage EC still undergo upfront surgery without prior neoadjuvant therapy. With advancements in early diagnostic technologies, an increasing number of EC cases are being detected at earlier stages^[31,32,33,34]^. This trend suggests that a growing number of patients with early-stage EC could benefit from the preventive effects of FS. On the other hand, the findings from this study are significant not only for the surgical management of EC but also for broader applications in benign esophageal disease such as esophageal atresia and caustic esophageal injury. Patients diagnosed with benign esophageal disease frequently undergo upfront esophagectomy, and the effectiveness of FS in preventing postoperative AL may be superior.

Our study demonstrated that FS can be safely applied intraoperatively, which aligns with findings from previous research^[16,35]^. There was no significant difference in postoperative complications between the two groups, with the exception of pneumonia and ARDS, which were both more frequent in the control group. Moreover, the number of lymph nodes dissected between the two groups was comparable, together with a similar incidence of recurrent laryngeal nerve injury, indicating that the quality of lymph node dissection between the two groups was equivalent.

This study has several limitations. All patients underwent McKeown esophagectomy with cervical anastomosis, and whether FS could prevent AL in patients who underwent Ivor Lewis esophagectomy with intrathoracic anastomosis still needs to be investigated in the future. Additionally, although chemoradiation remains the standard preoperative treatment for locally advanced EC, some patients with initially unresectable tumors received induction chemotherapy preoperatively. After chemotherapy, the tumors of these patients were subsequently deemed resectable by a multidisciplinary team.

In conclusion, the PLACE030 study provides high-level evidence that the application of FS did not significantly decrease the incidence of AL in patients who underwent McKeown esophagectomy. The unnecessary use of FS in EC patients who receive neoadjuvant treatment may impose an economic burden. However, the potential protective efficacy of FS in patients undergoing upfront esophagectomy warrants further investigation.

## Data Availability

The data used in this study can be made available upon reasonable request. Due to privacy and ethical estrictions, the data are not publicly accessible.

## Research Registration Unique Identifying Number (UIN)

This study is registered at https://clinicaltrials.gov (identifier: NCT03847857).
